# Supplementary material for: Impact of Nutritional Status on Clinical Outcomes of Patients Undergoing PRGF Treatment for Knee Osteoarthritis—A Prospective Observational Study
Source: Nutrients. 2025 Sep 30;17(19):3134. doi: 10.3390/nu17193134 (PMC12525571; doi:10.3390/nu17193134)
Supplement: Supplementary file 1 [file nutrients-17-03134-s001.zip › nutrients-3867975-supplementary.pdf]

ID	Glucose (mg/dl)	Creatinine (mg/dl)	Triglycerides (mg/dl)	Total Cholesterol (mg/dl)	HDL (mg/dl)	PCR (mg/dl)	HbA1c (mmol/mol)	AST (U/l)	ALT (U/l)	White blood cells*10^9/l	Red blood cells*10^9/l	Haemoglobin (g/dl)	Hematocrit %	Neutrophils %	Lymphocytes %	Monocytes %	Eosinophils %	Basophils %	Neutrophils *10^9/l	Lymphocytes *10^9/l	Monocytes *10^9/l	Eosinophils *10^9/l	Basophils*10^9/l	Platelets *10^9/l
REFERENCE RANGE	74-106	0,7-1,3	≤150	≤200	≥60	0-0,5	<40	3-34	10-49	3,98-10,04	3,93-5,22	11,2-15,7	34,1-44,9	/	/	/	/	/	1,56-6,13	1,18-3,74	0-0,80	0-0,36	0-0,80	182-369
1	102	0,79	227	147	33	0,08	40	20	24	8,24	4,66	15,2	35,8	50,5	40,8	7,4	0,8	0,5	4,16	3,36	0,61	0,07	0,04	255
2	95	1,05	164	254	47	0	33	31	55	7,02	5,62	16,5	34,7	62,5	29,5	6,7	1	0,3	4,39	2,07	0,47	0,07	0,02	209
3	119	0,93	56	162	65	0	36	17	14	6,12	4,48	14,3	34,6	64,9	21,4	10,6	2	1,1	3,97	1,31	0,65	0,12	0,07	218
4	79	0,81	67	195	114	0,23	37	27	23	8,41	4,85	14,3	33,6	52,8	37,1	9	0,6	0,5	4,44	3,12	0,76	0,05	0,04	146
5	89	0,98	113	246	54	0,1	36	30	36	10,28	5,35	16,8	34	59,6	22	6,8	10,3	1,3	6,13	2,26	0,7	1,06	0,13	334
6	98	0,88	387	288	46	0,65	38	18	12	11,06	3,98	12,5	33,1	57,6	30,2	10,8	0,9	0,5	6,37	3,34	1,19	0,1	0,06	294
7	94	1,33	149	247	58	0,18	39	18	21	8,53	4,54	14,7	33,8	50,6	41,7	6,1	0,8	0,8	4,31	3,56	0,52	0,07	0,07	310
8	112	0,97	176	247	42	0,05	37	41	66	8,55	5,75	15,7	32,5	54,6	34,2	10,4	0,1	0,7	4,67	2,92	0,89	0,01	0,06	205
9	101	0,77	70	236	65	0,05	36	20	23	6,24	4,84	13,2	32,2	48,3	42,3	6,9	1,9	0,6	3,01	2,64	0,43	0,12	0,04	264
10	87	0,99	153	209	40	0,08	35	34	55	5,26	5,25	13,3	34,2	50,9	36,9	8,9	2,3	1	2,68	1,94	0,47	0,12	0,05	372
11	90	0,87	70	155	36	0,05	32	22	16	7,06	5,06	15,1	34,6	61,3	28,2	7,5	2,4	0,6	4,33	1,99	0,53	0,17	0,04	185
12	77	1,02	98	166	39	0,18	35	30	31	7,6	5,26	15,8	35,5	54,5	35,4	7,5	2,2	0,4	4,14	2,69	0,57	0,17	0,03	288
13	68	0,68	72	183	62	0,08	34	26	25	4,77	4,34	13,2	32,5	80,5	10,7	6,3	1,7	0,8	3,84	0,51	0,3	0,08	0,04	229
14	88	1,25	309	258	62	0,08	38	18	19	8,75	5,46	15,8	33,9	54,9	34,6	8,1	1,8	0,6	4,8	3,03	0,71	0,16	0,05	213
15	74	1,05	103	239	68	0,05	37	19	21	6,61	4,71	14,8	33,9	54,4	32,4	8	3,3	0,9	3,66	2,14	0,53	0,22	0,06	319
16	81	0,99	104	163	55	0,05	36	25	19	5,16	4,86	14,4	30	61,5	29,7	6,8	1,2	0,8	3,18	1,53	0,35	0,06	0,04	184
17	\	0,84	57	183	75	0,05	33	26	15	6,84	5,15	15,4	31,2	67,2	25,1	6,1	0,7	0,9	4,59	1,72	0,42	0,05	0,06	241
18	78	1,25	274	248	40	0,06	34	25	31	8,89	5,34	16,4	30,7	69,6	21,7	7,2	1,1	0,4	6,18	1,93	0,64	0,1	0,04	322
19	86	1,54	102	231	51	0,05	35	22	16	7,15	4,84	14,4	34	76,5	14,4	8	0,8	0,3	5,47	1,03	0,57	0,06	0,02	174
20	87	0,74	53	183	69	0,07	32	20	19	5,42	3,77	12,2	33,6	40,5	50,4	6,5	0,9	1,7	2,2	2,73	0,35	0,05	0,09	336
21	72	0,71	91	209	68	0,05	32	21	12	6,89	4,82	14,1	33,2	59,6	32,2	6,5	1	0,7	4,1	2,22	0,45	0,07	0,05	308
22	72	1,27	139	208	56	0,18	36	22	18	6,5	5,11	14,1	31,8	55,6	32,6	6,8	4,5	0,5	3,62	2,12	0,44	0,29	0,03	241
23	77	1,09	81	204	50	0,05	33	27	23	6,89	5,1	16	34,8	66,6	20,6	7,4	5,1	0,3	4,59	1,42	0,51	0,35	0,02	186
24	81	0,72	262	204	52	0,05	34	21	20	7,73	6,24	18,8	32,2	60,5	27,4	7,4	3,9	0,8	4,68	2,12	0,57	0,3	0,06	186
25	83	0,97	129	222	57	0,05	34	20	19	6,56	6,02	18,6	33,2	44,2	41,9	9,3	3,7	0,9	2,9	2,72	0,61	0,24	0,06	231
26	85	0,6	402	262	47	0,14	28	20	18	5,94	4,75	14,8	34,3	63,3	25,4	6,6	4	0,7	3,76	1,51	0,39	0,24	0,04	218
27	92	0,95	125	241	50	0,05	35	19	24	9,72	4,87	14,3	33,6	62,9	28,7	6,2	1,9	0,3	6,12	2,79	0,6	0,18	0,03	207
28	94	0,65	189	255	71	0,07	35	17	14	6,58	4,59	13	32,2	60,6	27,4	10,3	1,4	0,3	3,99	1,8	0,68	0,09	0,02	314
29	83	1,01	77	199	56	0,05	29	21	19	6,12	4,61	14,5	34,3	57,5	30,7	8,2	2,8	0,8	3,52	1,88	0,5	0,17	0,05	196
30	86	0,77	62	180	53	0,14	32	18	18	7,97	4,76	14,1	33,1	60,8	25,8	7,4	5,1	0,9	4,84	2,06	0,59	0,41	0,07	266
31	69	0,79	74	169	61	0,22	37	18	14	5,86	3,84	12,5	32,5	55,9	28,7	12,1	2,4	0,9	3,28	1,68	0,71	0,14	0,05	247
32	86	0,68	89	216	66	0,05	35	21	19	9,05	5,05	13,6	32,8	51,5	38,3	8,5	1,4	0,3	4,65	3,47	0,77	0,13	0,03	223
33	79	0,82	104	263	67	0,2	34	32	26	5,47	4,92	15,4	34,4	56,7	34,2	7,1	1,5	0,5	3,1	1,87	0,39	0,08	0,03	213
34	92	0,78	67	155	57	0	41	15	15	5,01	4,88	14,5	32,6	43,7	42,1	11,2	2,2	0,8	2,19	2,11	0,56	0,11	0,04	284
35	88	1,25	62	202	61	0,27	37	30	59	8,3	5,36	15,8	33	66	21,2	10,5	1,7	0,6	5,48	1,76	0,87	0,14	0,05	232
36	84	0,84	57	156	52	0	33	17	20	6,68	5,07	15,2	34,2	65	25,9	7,5	1	0,6	4,34	1,73	0,5	0,07	0,04	231
37	81	0,89	51	230	82	0	34	34	44	5,22	5,21	14,7	33,3	58	28	6,7	6,3	1	3,03	1,46	0,35	0,33	0,05	189
38	80	0,84	79	181	46	0,44	42	20	17	6,19	4,79	13,8	32,9	53,5	35,7	6	4	0,8	3,31	2,21	0,37	0,25	0,05	296
39	91	1,31	122	256	47	1,16	38	20	21	8,07	5,16	15,2	33,2	61,5	27,9	7,6	2,4	0,6	4,97	2,25	0,61	0,19	0,05	325
40	87	0,86	194	102	102	0,45	39	26	29	5,76	4,93	13,2	31,7	52,3	36,6	6,9	3	1,2	3,01	2,11	0,4	0,17	0,07	203
41	83	0,82	76	189	63	0,14	37	23	20	4,79	4,69	14,3	32,6	40,4	47,2	9	2,1	1,3	1,94	2,26	0,43	0,1	0,06	207

Supplementary Table S1: Complete blood count, Glucose, Creatinine, Triglycerides, Total Cholesterol, High-density Lipoproteins (HDL), C-Reactive Protein (CRP), Glycated Hemoglobin (A1c), Aspartate Aminotransferase (AST)

and Alanine aminotransferase (ALT) values, with their respective normal reference ranges of all included patients (N=41).

ID	PRE-TREATMENT						2 MONTHS FOLLOW-UP						6 MONTHS FOLLOW-UP						12 MONTHS FOLLOW-UP					
	KOOS TOT	KOOS PAIN	KOOS SYMTOMPS	KOOS ACTIVITY	KOOS SPORT	KOOS qol	KOOS TOT	KOOS PAIN	KOOS SYMTOMPS	KOOS ACTIVITY	KOOS SPORT	KOOS qol	KOOS TOT	KOOS PAIN	KOOS SYMTOMPS	KOOS ACTIVITY	KOOS SPORT	KOOS qol	KOOS TOT	KOOS PAIN	KOOS SYMTOMPS	KOOS ACTIVITY	KOOS SPORT	KOOS qol
1	74.40	38.9	64.28	42.64	10	12.5	/	/	/	/	/	/	76.78	77.77	85.71	79.41	70	56.25	82.73	86.11	82.14	86.76	12	75
2	78.57	83.33	67.86	94.12	75	25	64.28	66.7	53.57	94.11	20	6.25	/	/	/	/	/	/	/	/	/	/	/	/
3	72.62	75	75	86.76	45	37.5	91.66	94.44	92.85	98.52	85	62.5	/	/	/	/	/	/	95.83	97.22	92.85	100	100	75
4	60.71	61.11	71.42	70.58	35	31.25	57.14	61.11	75	63.23	30	25	64.28	63.88	78.57	69.11	45	43.75	62.5	61.11	75	63.23	45	62.5
5	81.55	94.4	78.57	83.82	70	62.5	95.83	97.22	96.42	98.52	90	87.5	88.09	94.44	92.85	89.7	80	68.75	/	/	/	/	/	/
6	45.24	50	53.57	66.17	40	18.75	89.28	91.66	78.57	97.05	75	87.5	/	/	/	/	/	/	/	/	/	/	/	/
7	84.52	9	92.85	95.58	65	31.25	84.52	94.44	96.42	91.17	65	37.5	82.73	86.11	82.14	89.7	70	62.5	88.69	100	82.14	95.58	80	56.25
8	68.45	72.22	75	86.76	30	18.75	62.5	52.77	75	77.94	35	31.25	/	/	/	/	/	/	/	/	/	/	/	/
9	69.05	63.88	75	85.29	30	50	87.5	83.33	89.28	97.05	70	75	84.52	77.77	85.71	94.11	80	62.5	89.28	91.66	92.85	95.58	70	75
10	83.93	83.33	78.57	100	80	31.25	88.69	86.11	92.85	97.05	80	62.5	/	/	/	/	/	/	/	/	/	/	/	/
11	82.74	83.33	78.57	95.58	65	56.25	84.52	86.11	89.28	92.64	65	62.5	90.47	88.88	96.42	97.05	75	75	/	/	/	/	/	/
12	39.29	38.9	50	50	5	18.75	70.23	69.44	71.42	80.88	50	50	84.52	88.88	85.71	92.64	55	75	93.45	94.44	82.14	98.52	95	87.5
13	71.43	69.44	85.71	77.94	60	37.5	86.3	80.55	89.28	94.11	85	62.5	70.02	63.88	85.71	75	65	62.5	97.02	97.22	100	97.05	90	100
14	70.24	75	71.42	76.47	50	56.25	94.64	94.44	96.42	97.05	95	81.25	/	/	/	/	/	/	/	/	/	/	/	/
15	75.00	77.77	64.28	91.17	50	50	72.02	72.22	60.71	83.82	55	62.5	86.3	86.11	75	95.58	80	75	/	/	/	/	/	/
16	79.17	72.22	78.57	98.53	55	43.75	79.16	80.55	75	98.52	55	31.25	75	75	78.57	94.11	40	31.25	77.38	77.77	78.57	98.52	35	37.5
17	76.79	75	57.14	97.05	75	31.25	/	/	/	/	/	/	/	/	/	/	/	/	/	/	/	/	/	/
18	75.60	63.88	82.14	94.11	45	50	92.85	83.33	92.85	98.52	100	81.25	67.26	61.11	78.57	77.94	35	56.25	/	/	/	/	/	/
19	83.33	86.11	82.14	97.05	70	37.5	68.45	61.11	67.85	69.11	100	43.75	82.14	83.33	89.28	85.29	90	43.75	73.8	77.77	75	85.29	55	37.5
20	63.10	66.7	46.42	85.29	25	37.5	/	/	/	/	/	/	77.38	77.77	75	94.11	40	56.25	/	/	/	/	/	/
21	36.90	36.11	21.42	57.35	0	25	61.3	66.66	60.71	77.94	0	56.25	73.8	80.55	67.85	86.76	25	75	82.14	86.11	67.85	89.7	75	75
22	61.90	63.88	71.42	70.58	30	43.75	/	/	/	/	/	/	60.71	61.11	75	66.17	30	43.75	/	/	/	/	/	/
23	43.45	52.77	57.14	48.52	15	12.5	66.01	72.22	71.42	88.23	15	12.5	/	/	/	/	/	/	/	/	/	/	/	/
24	51.79	52.77	64.28	60.29	30	18.75	58.33	75	71.42	58.82	40	18.75	73.8	83.33	67.85	89.7	65	6.25	95.23	100	92.85	98.52	100	68.75
25	49.40	38.88	67.85	64.7	5	31.25	55.95	55.55	71.42	55.88	45	43.75	58.92	63.88	75	64.7	20	43.75	63.09	80.55	71.42	58.82	45	50
26	45.83	52.77	39.28	64.7	5	12.5	77.38	80.55	75	91.17	45	56.25	/	/	/	/	/	/	55.95	63.88	46.42	72.05	10	43.75
27	61.90	61.11	57.14	80.88	30	31.25	72.02	77.77	57.14	91.17	40	43.75	75.59	72.22	78.57	86.76	75	31.25	59.52	63.88	78.57	63.23	45	18.75
28	80.36	80.55	92.85	82.35	75	56.25	69.64	75	71.42	75	55	50	/	/	/	/	/	/	/	/	/	/	/	/
29	78.57	75	85.71	97.05	45	37.5	67.85	69.44	82.14	83.82	20	31.25	/	/	/	/	/	/	74.4	77.77	92.85	83.82	45	31.25
30	76.79	75	85.71	80.88	65	62.5	72.02	77.77	82.14	76.47	50	50	68.45	69.44	75	82.35	30	43.75	70.83	77.77	85.71	80.88	25	43.75
31	50.60	58.33	53.57	66.17	5	18.75	71.42	66.7	67.85	80.88	75	43.75	77.38	86.11	78.57	83.82	60	50	56.54	50	57.14	54.41	90	37.5
32	13.10	16.66	32.14	4.41	0	25	/	/	/	/	/	/	/	/	/	/	/	/	/	/	/	/	/	/
33	90.48	91.66	85.71	98.52	85	68.75	86.3	80.55	89.28	94.11	90	56.25	/	/	/	/	/	/	/	/	/	/	/	/
34	76.19	72.22	85.71	85.29	60	50	/	/	/	/	/	/	/	/	/	/	/	/	/	/	/	/	/	/
35	60.12	63.88	82.14	70.58	20	18.75	74.4	77.77	82.14	88.23	45	31.25	80.95	88.88	92.85	89.7	60	31.25	/	/	/	/	/	/
36	64.29	77.77	67.85	70.58	45	25	60.71	63.88	78.57	61.76	55	25	59.52	66.66	60.71	63.23	45	43.75	/	/	/	/	/	/
37	70.83	72.22	64.28	86.76	40	50	77.38	75	75	91.17	50	62.5	75.59	80.55	67.85	86.76	50	62.5	79.16	88.88	60.71	95.58	45	62.5
38	71.43	80.55	82.14	79.41	35	43.75	96.42	97.22	96.42	100	85	93.75	78.57	86.11	85.71	83.82	45	68.75	91.66	94.44	92.85	92.64	75	100
39	79.76	91.66	82.14	89.7	60	31.25	93.45	97.22	89.28	98.52	100	62.5	89.88	97.22	82.14	98.52	80	62.5	/	/	/	/	/	/
40	75.60	72.22	78.57	91.17	50	43.75	77.38	77.77	82.14	86.76	60	50	91.66	94.44	96.42	98.52	80	75	89.88	97.22	85.71	97.05	80	62.5
41	58.93	52.77	35.71	82.35	40	37.5	66.7	61.11	42.85	89.7	35	62.5	74.4	75	50	95.58	45	62.5	67.85	63.88	53.57	88.23	30	62.5

Supplementary Table S2: KOOS Total and KOOS Subscale scores at pre-treatment. 2. 6 and 12 months follow-up

A

CD Marker	*p*-value	Adjusted *p*, *q*-value
CD86	0.0356	0.096 #
CD1c	0.059	0.096 #
CD4	0.0655	0.096 #
CD19	0.0767	0.096 #
CD20	0.0767	0.096 #
CD44	0.089	0.096 #
MCSP	0.096	0.096 #

B

CD Marker	*p*-value	Adjusted *p*, *q*-value
MCSP	0.0514	0.0826 #
CD19	0.0514	0.0826 #
CD20	0.0605	0.0826 #
CD86	0.0708	0.0826 #
CD11c	0.0901	0.0901 #

Supplementary Table S3: Tendency relative to EV's markers in responder *vs* non-responder patients at two **(A)** and twelve **(B)** months follow up
